# Intra-arterial adenoviral mediated tumor transfection in a novel model of cancer gene therapy

**DOI:** 10.1186/1476-4598-5-32

**Published:** 2006-08-09

**Authors:** Gustavo Cabrera, Stacy L Porvasnik, Paul E DiCorleto, Maria Siemionow, Corey K Goldman

**Affiliations:** 1 Gene Therapy Laboratory, National Cancer Institute, Mexico City, Mexico; 2 Powel Gene Therapy Center, The University of Florida, Gainesville, USA; 3 Department of Cell Biology, Lerner Research Institute, The Cleveland Clinic Foundation, Cleveland, USA; 4 Department of Plastic and Reconstructive Surgery, The Cleveland Clinic Foundation, Cleveland, USA; 5 Department of Vascular Medicine, Ochsner Clinic Foundation, New Orleans, USA

## Abstract

**Background:**

The aim of the present study was to develop and characterize a novel *in vivo *cancer gene therapy model in which intra-arterial adenoviral gene delivery can be characterized. In this model, the rat cremaster muscle serves as the site for tumor growth and provides convenient and isolated access to the tumor parenchyma with discrete control of arterial and venous access for delivery of agents.

**Results:**

Utilizing adenovirus encoding the green fluorescent protein we demonstrated broad tumor transfection. We also observed a dose dependant increment in luciferase activity at the tumor site using an adenovirus encoding the luciferase reporter gene. Finally, we tested the intra-arterial adenovirus dwelling time required to achieve optimal tumor transfection and observed a minimum time of 30 minutes.

**Conclusion:**

We conclude that adenovirus mediated tumor transfection grown in the cremaster muscle of athymic nude rats *via *an intra-arterial route could be achieved. This model allows definition of the variables that affect intra-arterial tumor transfection. This particular study suggests that allowing a defined intra-tumor dwelling time by controlling the blood flow of the affected organ during vector infusion can optimize intra-arterial adenoviral delivery.

## Background

The therapeutic efficacy of novel cancer therapeutic agents including genetic vectors that directly target tumor cells greatly depends on their adequate distribution to and within the tumor mass [1-6]. The capacity of adenoviral vectors to reach and transfect the highest possible number of cells that constitute the tumor mass is thus critical for the success of adenoviral cancer gene therapy [5,6]. Various routes and methods to deliver genetic vectors to the tumor mass have been used [7-9]. The route and method chosen may have a profound effect on tumor transfection efficiency and thus on therapeutic efficacy. Direct intra-arterial delivery of vector has also been utilized and represents a viable clinical and experimental route [10-16]. Other delivery methods employed include intraperitoneal, intra-tumor, intravenous and intravesical administration routes [7-16]. While some clinical trials have been positive, further improvement should include a systematic assessment of the variables that affect intra-arterial tumor transfection. To date, substantial work has been done to understand the variables that affect tumor drug bioavailability [1-4]. Three properties of tumors result in poor distribution of macromolecules in tumors: 1) a heterogeneous disposition of blood vessels within the tumor; 2) elevated tumor interstitial pressure; and 3) large transport distances in the tumor interstitium [1-4]. Various in vivo models have been used to characterize tumor vasculature and macromolecular tumor dynamics [17,18]. Among these, the rat cremaster muscle has been used as a model to study microcirculatory hemodynamics in various pathologic and physiologic conditions [19-22]. The rat cremaster muscle has three important properties that make it an attractive site for the growth of solid tumors and to study the dynamics involved in the intra-arterial delivery of gene vectors for cancer gene therapy. Firstly, the rat's cremaster muscle is fed by one principal artery and drained by one main vein [23]. Secondly, the microsurgical dissection and manipulation of these vessels enable simultaneous access to the tumor site *and *vascular inflow and outflow. Thirdly, the cremaster muscle is constituted by well-vascularized skeletal muscle that provides a useful substrate for the growth of tumor masses. We thus tested the hypothesis that tumor masses grown on the cremaster muscle of male athymic nude rats could be transfected via the intra-arterial route. In these studies, we describe the tumor cremaster model and demonstrate the time and viral particle number dependence for adenoviral gene transfer.

## Results

### Tumor growth on the cremaster of homozygous athymic male nude rats

**Figure 1:**
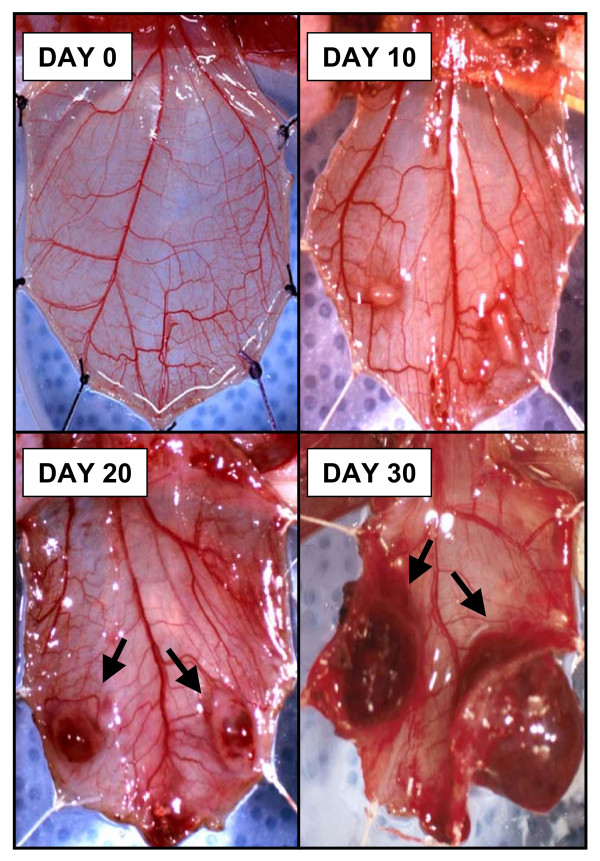
**Tumor growth on the cremaster muscle of athymic nude male rats**. The cremaster muscle of athymic male nude rats were inoculated with 5 × 10^4 ^cells of the human bladder carcinoma cell line T24. Tumor growth was followed at days 10, 20 and 30 post cell injection.

**Figure 2:**
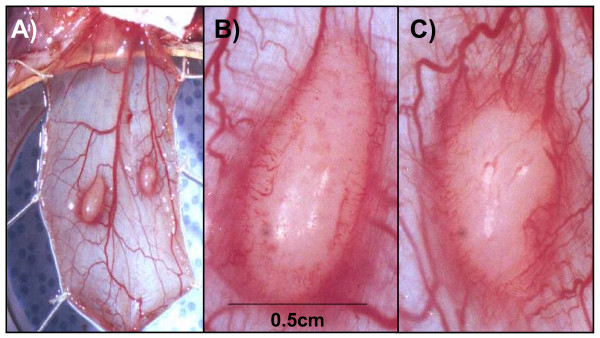
**Angiogenic response elicited by tumor masses growing in the cremaster muscle of athymic nude rats**. The cremaster muscle of athymic male nude rats were inoculated with 5 × 10^4 ^human T24 bladder carcinoma cells. Ten days after tumor cell inoculation rats were sacrificed and mounted for photographic documentation. Concurrent with tumor growth we observed recruitment of new blood vessels that grew in a concentric pattern. Peri-tumoral edema characteristic of tumor growth can be observed. A. 1× magnification of the cremaster muscle bearing tumors on both sides of the principal artery, B. 2× magnification of left tumor, C. 2× magnification of the right tumor.

We initially tested if a tumor mass could be grown in the cremaster muscle of athymic male nude rats by inoculating 5 × 10^4 ^cells of the human bladder carcinoma cell line T24 into the muscle and documented tumor growth at days 10, 20 and 30 post cell injection. As seen in Figure 1, the tumor masses grew beyond 5 mm in diameter. Concurrent with tumor growth we observed recruitment of new blood vessels. As shown in Figure 2, we observed concentric growth of blood vessels that infiltrate the tumor as well as peritumoral edema.

### Spatial distribution of intra-arterial adenoviral mediated tumor transfection

**Figure 3:**
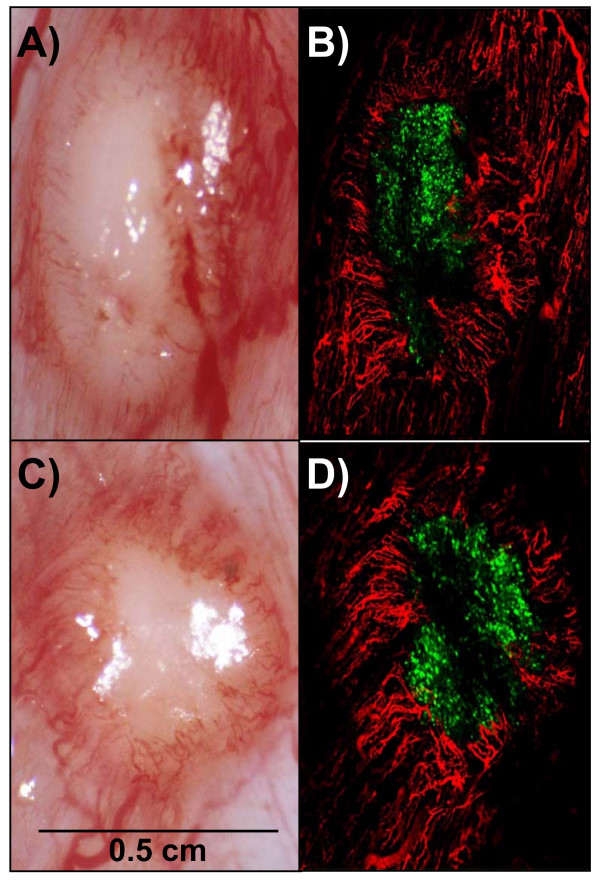
**Intra-arterial adenoviral mediated transfection of tumors grown in the cremaster muscle of athymic nude rats**. Tumors from two different animals are shown. The cremaster muscle of athymic male nude rats were inoculated with 5 × 10^4 ^cells of the human bladder carcinoma cell line T24. Ten days after tumor cell implantation, the cremaster muscle flap was intra-arterially infused with 1 × 10^9 ^pfus of a recombinant adenovirus encoding the green fluorescent protein gene (Ad5-CMV-GFP) and were left to incubate for 1 hour. Seventy two hours after viral infusion cremasters were set up for standard micro-photography and fluorescent confocal microscopy and photography as described in materials and methods. Texas red fluorescent micro beads were used to counter stain the muscle and tumor vasculature. Panel A: 2X Photographic image of a tumor using a standard stereoscopic microscope. Panel B Confocal fluorescent photography of the same tumor. Panel C: 2X Photographic image of a tumor using a standard stereoscopic microscope on a second animal. Panel D: Confocal fluorescent photography of the same tumor. Green fluorescent protein can be seen expressed throughout the tumor mass. A concentric neoangiogenic response can be seen as well.

We then studied if tumors grown in the cremaster muscle of athymic nude rats could be transfected using recombinant adenoviral vectors via the intra-arterial route. Cremaster muscle bearing tumor was transfected with Ad5-CMV-GFP for 1 hour and examined for the presence and distribution of the expressed reporter green fluorescent protein (GFP) within the tumor mass seventy-two hours post Ad5-CMV-GFP transduction. The tumor vasculature was counterstained using intra-arterial infusion of red fluorescent 1 micron diameter beads and mounted for fluorescent confocal microscopy as described in material and methods. As seen in Figure 3, panels B and D, confocal microscopy revealed diffuse transfection of the tumor mass. A concentric neo-angiogenic response elicited within the tumor mass was also observed. Muscle transfection was also observed throughout the muscle pedicle (data not shown).

### Dose dependence of in vivo adenovirus gene transfection

**Figure 4:**
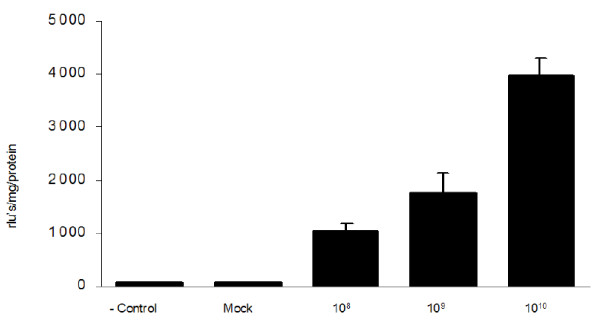
**Dose curve effect on adenoviral mediated intra-arterial tumor transfection**. Tumor bearing animals were infused with 1 × 10^8 ^(n = 8), 1 × 10^9 ^(n = 8) and 1 × 10^10 ^(n = 8) of Ad-CMV-Luc using the surgical approach described in materials and methods. After intra-arterial adenoviral infusion was completed, the cremasters bearing tumors were left to incubate for 1 hour. After the time had elapsed, clamps were released and rats were left to live for 72 hrs. The rats were then sacrificed and the samples were assayed for luciferase activity as described in materials and methods. The results show dose curve dependant luciferase activity at the tumor site.

Once we visualized the spatial distribution of green fluorescent reporter gene expression in tumors we used a similar recombinant adenovirus carrying the Luc reporter gene (Ad-CMV-Luc) to quantify the profile of tumor transfection. The data presented in Figure 4 demonstrate increasing luciferase activity in the tumor mass after in vivo adenoviral transfection using doses of 1 × 10^8^, 1 × 10^9 ^1 × 10^10 ^plaque forming units (PFU's) in a fixed volume of 200 microliters. Each viral transfection was performed with a vascular isolation-adenovirus dwelling time of 60 minutes.

### Temporal dependence on incubation time for adenoviral mediated intra-arterial tumor transfection

In the initial experiments the cremaster vascular supply isolation time in the presence of adenovirus was 60 minutes. This time frame was chosen based on standard ex vivo adenoviral transfection protocols in which adenovirus is allowed to be in contact with the target cells for no less than 60 minutes [22,23]. In an applied in vivo clinical scenario, arterial blood flow interruption would be kept to a minimum yet maximum tumor transfection would be desired. We sought to determine a minimum intra-cremasteric virus dwelling time required to achieve acceptable reporter gene transfection with 60 minutes being the maximal blood interruption time. Two shorter durations of vascular isolation were assessed; 5 minutes and 30 minutes. To this end, tumor-bearing animals were infused with Ad-CMV-Luc using the surgical method described previously. After the specified dwelling time had elapsed, arterial and venous circulation was re-established and the samples were processed as described in materials and methods. The data presented in Figure 6 suggest that an intra-cremaster dwelling time of 5 minutes yields 21% transfection efficacy when compared to the 60 minute group while an intracremaster adenovirus dwelling time of 30 minutes yielded a 91% transfection efficacy when compared to the 60 minute group. No statistical significance was observed between the 30 minute group and the 60 minute group (p > 0.05).

## Discussion

**Figure 5:**
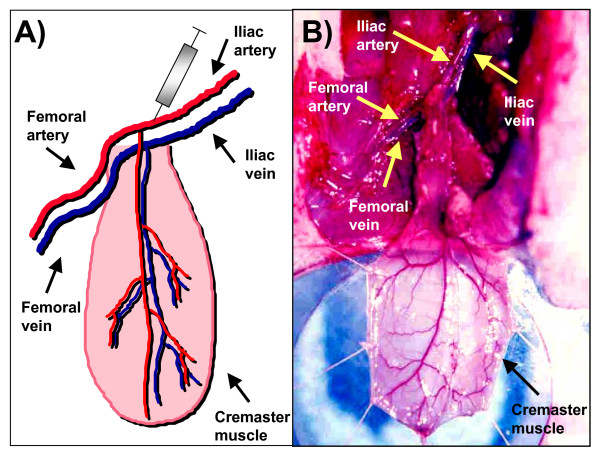
**Anatomy of the cremaster muscle in the athymic male nude rat**. A. Diagram depicting the blood vessel anatomy and the intra-arterial access to the cremaster muscle and the tumor site. B. Actual anatomy of the vessels that feed and drain the cremaster muscle of male rat.

**Figure 6:**
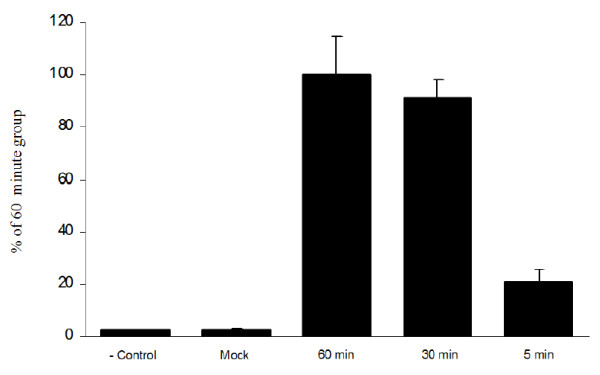
**Effect of incubation time on adenoviral mediated intra-arterial tumor transfection**. Cremaster tumor bearing animals were infused with 1 × 10^10 ^Ad-CMV-Luc using the surgical approach described in materials and methods. After intra-arterial adenoviral infusion was completed, the cremasters bearing tumors were left to incubate for either 5 minutes (n = 8), 30 minutes (n = 8) or 60 minutes (n = 8). After the times had elapsed, clamps were released and rats were left to live for 72 hrs. The rats were then sacrificed and the samples were processed as described in materials and methods. The data presented suggest that intra-arterial adenoviral mediated tumor transfection could be achieved optimally with a dwelling time of 30 minutes.

The efficacy of novel cancer treatment modalities including the use of gene therapy strategies depends on their ability to deliver genetic information to the tumor mass in adequate quantities [1-6]. In general, cancer gene therapy strategy success greatly depends on achieving high tumor transfection profiles in addition to an effective anticancer strategy [5,6]. In human and animal studies, an intra-arterial route of gene therapy vector administration represents a viable method in order to transfect the tumor parenchyma and normal tissue [9-16]. The lack of success of some intra-arterial gene therapy trials may be related to delivery issues rather than payload bioactivity. A systematic assessment of the variables that affect tumor transfection via the intra-arterial route in a cancer gene therapy context would be useful to refine the delivery technique to accomplish efficient tumor vector expression. In this regard, substantial research has been done to define the variables that optimize molecular bioavailability [1-4,17]. For a blood-borne therapeutic agent to reach the neoplastic cells, it must enter the blood circulation, cross the vessel wall, move across the extracellular matrix, and finally reach the tumor cell [1-4]. In addition to these anatomic impediments, three physiological barriers are responsible for the poor bioavailability of macromolecules in tumors: 1) a heterogeneous tumor vascular supply limits the delivery of therapeutic molecules to well vascularized or well-perfused regions of the tumor; 2) elevated tumor interstitial pressure reduces the penetration of the therapeutic macromolecules whereby a high pressure outward radial gradient leaves tumor regions without drug; and 3) large transport distances within the tumor interstitium slow down the transit of the macromolecules and thus make distal regions of a tumor hard to reach [1-4]. The rat cremaster muscle has been extensively used to study tumor molecular dynamics, structural properties of the tumor vasculature and microcirculatory dynamics of various pathophysiologic conditions [18-23]. In addition, the rat cremaster muscle has several properties that make it an attractive site for the growth of tumors and for its use as an endovascular cancer gene therapy model. As seen in Figure 5, the cremasters vascular anatomy enables the surgical dissection, manipulation and isolation of the iliac and common femoral arteries and veins which permits the direct infusion of agents into the pudo-epigastric artery that feeds the cremaster muscle. Another desirable property of the cremaster muscle relates to well vascularized skeletal muscle. This is essential for growth of vascularized tumor masses and thus to access the tumors parenchyma *via *the intra-arterial route. Finally, the inside aspect of the cremaster tube muscle provides a protected encased environment in which the tumors can grow. As seen in Figures 1 and 2, the careful surgical manipulation of the cremaster after several weeks of tumor growth did not damage the integrity of the tumor mass, the tumor vasculature or the surrounding tissue. This issue is of relevance if further measurements such as microcirculatory hemodynamics, tumor dimensions and tumor vasculature measurements are to be done. In order to characterize a novel in vivo intravascular cancer gene therapy model we initially tested if a tumor mass could be grown in the cremaster muscle of athymic male nude rats and if the masses grew beyond a few millimeters. The growth of tumors beyond a few millimeters is relevant for two main reasons: 1) Growth beyond 2 mm in diameter requires neo-angiogenesis and thus enables the intravascular access to the tumors parenchyma [24] and 2) growth beyond several millimeters in diameter during a period of 30 days would allow the model to be used in anti-cancer studies. Additionally, the need to test the therapeutic efficacy of emerging anti-cancer agents on tumor masses larger than a few millimeters in diameter for tumor regression studies has been stated [25]. Vascularized tumor masses developed in the cremaster of homozygous athymic male nude rats and grew beyond a few millimeters as seen in our studies (Figure 1). Once established that vascularized tumor masses could be generated, we wanted to determine if we could accomplish intra-arterial tumor transfection using adenoviral vectors. We therefore examined the presence of the expressed green fluorescent protein in the tumor mass. Confocal microscopy revealed diffuse transfection of the tumor mass. As seen in Figure 3 panels A-D a concentric neo-angiogenic response was observed using stereoscopic microscopy and when the vasculature was counterstained with red fluorescent microbeads. Muscle transfection was also observed throughout the muscle pedicle (data not shown). We then tested the dose dependence effect on tumor transfection using an adenovirus encoding the Luciferase reporter gene (Ad-CMV-Luc). The data presented in Figure 4 indicate that in vivo transfection of the tumor mass via the intra-arterial infusion of recombinant adenoviral vectors was achieved as reflected by the presence of luciferase activity in the tumor masses. The observed increment in luciferase activity within the tumors correlates with the incremental doses of adenovirus infused. As demonstrated by clinical trials and various animal models, intra-arterial delivery of genetic vectors to the cancer affected organ remains a viable route [9-16]. In the present model, we initially tested Ad5-CMV-GFP and Ad-CMV-Luc tumor transfection by closing the vascular circuitry of the cremaster muscle for 60 minutes, thus allowing the virus to bind to its receptor and internalize. This time frame was utilized based on standard adenoviral protocols in which the studied cells are allowed to be in contact with adenovirus for no less than 60 minutes [26,27]. In a clinical scenario the interruption of arterial blood flow to a given organ for 60 minutes would not seem optimal due to prolonged anoxia. Arterial blood flow interruption of the target organ would thus have to be kept to a minimum yet enough time could be allotted in order to allow maximum tumor transfection. We thus tested how varying the intra cremaster virus dwelling time affected tumor transfection. We selected three incubation times; 5 minutes, 30 minutes and 60 minutes and an adenoviral dose of 1 × 10^10^. As seen in Figure 6, allowing the virus to dwell within the cremaster for 30 minutes yielded transfection profiles similar to the 60 minute incubation time group. Arterial blood flow interruption for a period of 30 minutes may be viable for various tumor types since the treated normal tissues would be exposed to decreased anoxia time and still allow adenoviral binding and internalization. An intra- cremaster dwelling time of 5 minutes revealed relatively low adenoviral transfection of the tumor mass when compared to the 30- and 60-minute incubation times. These findings have implications for anoxia sensitive organs including the kidney and brain whereby a 30-minute in vivo occlusion time is unlikely to be acceptable, and thus the optimal virus dwelling time to achieve optimal viral transfection should be defined on a per organ basis. In addition to the three physiologic barriers that affect macromolecular tumor transport discussed above, optimal virus-tumor interaction time should be defined if therapeutic tumor transfection efficiency is to be achieved. Additional research needs to be done in order to study the variables, conditions, and mechanics such as temperature and injection pressure in the intra-arterial route in order to accomplish the optimal delivery. Our results suggest that the intra-organ virus dwelling time is a relevant variable that has an influence on tumor transfection efficiency in vivo.

## Conclusion

Our study demonstrates the in vivo dependence of adenoviral contact (dwell) time and dosage on transfection. A novel model is also described that could be of utility to further study variables that affect vector delivery to the tumor mass via the intra-arterial route and thus refine the optimal conditions to enhance tumor transfection. Finally, the present model could have utility to test validation of emerging anti-tumor vasculature or anticancer agents employing in vivo tissue targeting and differential vascular or tissue toxicities relative to the hosts normal vasculature and tissue.

## Methods

The Internal Review Board and Animal Research Committee of the Cleveland Clinic Foundation approved all animal experiments. In addition, all animals used in this study received the humane care in compliance with the Guide for the Care and Use of Laboratory Animals published by the National Institutes of Health.

### Animals

Athymic homozygous male nude rats with a weight of 100 – 110 gm were obtained from Harlan Sprague Dawley Inc. (Indianapolis, IN). Animals were kept under standard rodent laboratory housing conditions with 12 hours day/night cycles and given standard rodent chow diets (Nutrition International Inc., Brentwood, MO) and water ad libitum.

### Cell lines

The human bladder carcinoma cell line T24 was obtained from American Type Culture Collection (Rockville, MD). The cell line was grown in McCoys 5a medium (Sigma, St. Louis, MO) supplemented with 10% heat-inactivated fetal bovine serum (Hyclone, Logan, UT) and 2 mmol/L-glutamine, 100 U/mL penicillin G sodium, streptomycin sulfate, 0.25 g/mL.

### Adenoviral vectors

Ad5-CMV-GFP encodes the green fluorescent protein (GFP) gene driven by the cytomegalovirus promoter obtained from Q-Biogene Inc. (Montreal, Canada). The adenovirus Ad-CMV-Luc encodes the luciferase gene driven by the cytomegalovirus (CMV) promoter and was a kind gift from Dr. David Curiel at the University of Alabama at Birmingham. Adenoviral preparations and titering were performed as previously described (7).

### Tumor cell inoculation into the cremaster muscle

Tumor cell inoculation into the cremaster muscle was done under general anesthesia with an intraperitoneal injection of sodium pentobarbital (50 mg/kg) (Abbott Laboratories, Chicago, IL). The skin was diagonally incised from the middle of the scrotum to the inguinal ligament. The cremaster muscle was dissected free from the scrotum as previously described [23]. Briefly, the testis and spermatic cord were freed from the interior of the muscle tube flap through a horizontal incision in the anterior surface of the cremaster muscle at the level of the inguinal ring and were subsequently guided back into the abdominal cavity. The muscle tube was left inverted for tumor cell inoculation. Under microsurgical observation (Zeiss S3 OPMI operating microscope, Carl Zeiss, Gottingen, Germany), 5 × 10^4 ^human T24 bladder carcinoma cells resuspended in 50 μl of phosphate buffer saline (PBS) solution (Long Island City, GIBCO) were injected into the cremaster muscle wall using a 1 mL insulin syringe (Becton & Dickinson Corp. Franklin, NJ) with a 30 gauge 1/2 inch needle (Becton & Dickinson Corp.). Two intramuscular inoculations, one on either side of the main artery and vein of the muscle tube flap were performed on the right cremaster muscles. Subsequently, the cremaster muscle tube was returned to its normal anatomical position and placed back in the scrotal bag. The skin was sutured with 5-0 Vicryl (Ethicon Inc. Somerville, NJ) and the animals were given antibiotics (5,000 IU/kg subcutaneous) Penicillin G Benzathine and Penicillin G Procaine (G.C. Hartford Mfg. Corp., Syracuse, NY), 5 ml/S.C. of Ringer's solution (Baxter Corp. Dearfield, IL) and analgesics, Acetaminophen 110 mg/kg/PO (McNeil-PCC Inc., Fort Washington, PA). For the tumor growth curve, animals were sacrificed at days 10, 20 and 30 following tumor cell implantation by an intraperitoneal overdose of sodium pentobarbital. Collected tumor samples were measured, representative photographs were taken and the tumors were then sectioned and counter stained with hematoxylin and eosin for histopathologic evaluation.

### Intra-arterial delivery of recombinant adenoviral vectors

Intra-arterial infusion of the viral vectors was performed 10 days following tumor inoculation. Under sodium pentobarbital anesthesia, the previously placed skin sutures were carefully cut and the cremaster muscle was dissected free from the scrotum. The cremaster muscle flap and its supplying pudo-epigastric vascular pedicle were dissected to its origin at the iliac vessels. The external iliac artery, the proximal femoral artery and vein were clamped with microsurgical aneurysm clamps (Accurate Surgical & Scientific Instruments Corp., Westbury, NY) to create a cremaster muscle end-organ tube flap. Using a 1 mL tuberculin syringe with a 30 gauge 1/2 inch needle, the cremaster muscle tube flap was primed with 200 μl of PBS solution *via *the external iliac artery.

### Ad5-CMV-GFP infusion and confocal microscopy

For the intra-arterial delivery of recombinant adenovirus encoding the green fluorescent protein (Ad5-CMV-GFP), 1 × 10^9 ^pfu's in a total volume of 200 μl using a 1 mL tuberculin syringe with a 30 gauge 1/2 inch needle were used in all animals (n = 4). Briefly, the femoral artery was clamped with microsurgical aneurism clamps in order to direct the infused solution into the pudo-epigastric artery as shown in Figure 5, panel A. The iliac artery was also clamped to stop arterial blood flow and allow injection into the artery to proceed without bleeding. Immediately after adenoviral infusion was completed, the external iliac vein was clamped to avoid retrograde venous blood flow into the muscle flap. The puncture site at the iliac artery was sutured with 10-0 nylon microsurgical suture (Surgical Specialities Corp., Reading, PA). The muscle flaps were then left to incubate for 1 hour wrapped with a moist gauze. After 1 hour had elapsed, clamps were removed, circulation into the cremaster muscle flap was re-established and the muscle tube was inserted into a subcutaneous tunnel in the anteromedial aspect of the ipsilateral limb and the animals were returned to their cages for observation.

### Confocal microscopy

Seventy-two hours following Ad5-CMV-GFP infusion, animals were sacrificed with an intraperitoneal overdose of sodium pentobarbital and the cremaster muscle tube flap was carefully withdrawn from the subcutaneous tunnel of the limb. A round flat muscle flap with an axial pattern of vessels was created by vertically transecting the frontal wall of the cremaster muscle tube from the inguinal ring to the tip of the tube using a thermal cautery. The animal was secured on a specially designed Plexiglass tissue bath as previously described [10] and the cremaster muscle was spread out with 5-0 Ethibond (ETHICON Inc.) sutures over a cover glass. The external iliac artery and the proximal femoral artery and vein were dissected and clamped with microsurgical aneurism clamps isolating the vascular access into the muscle flap. Using a 1 mL insulin syringe with a 30 gauge 1/2 inch needle, the cremaster muscle tube flap was primed with 200 μl of PBS solution via the external iliac artery. Subsequently, 200 μl of a solution containing 1- micron diameter Texas Red fluorescent microbeads (Molecular Probes Inc., Eugene, OR) were infused using a 1 mL insulin syringe with a 24 gauge 0.75 inch Angiocath. This permitted red counter staining of the cremaster and tumor vasculature. After fluorescent microbead infusion was completed, the cremaster muscle pedicle was ligated at its iliac vessel origin with 5-0 Ethibond (ETHICON) and transected with a thermal cautery. The free flat cremaster muscle flap was then fixed with a 10% buffered formalin solution, for 1 hour and was placed on a tissue slide for confocal microscopy. Confocal microscopy images were collected using a Leica TCS-NT laser scanning spectrophotometric confocal microscope (Leica Microsystems AG, Mannheim, Germany).

### Adenoviral dose curve effect

For the dose curve intra-arterial delivery of recombinant adenovirus encoding the luciferase reporter gene (Ad-CMV-Luc) experiments, three groups of cremaster tumor bearing rats were administered the following doses of Ad-CMV-Luc; 1 × 10^8 ^(n = 8), 1 × 10^9 ^(n = 8) and 1 × 10^10 ^(n = 8) following the surgical procedure described above. Seventy two hours following intra-arterial Ad-CMV-Luc delivery, animals were sacrificed with an intraperitoneal overdose of sodium pentobarbital. Tumors were collected in 1.5 mL polypropylene tubes and luciferase assay was performed as described below.

### Intra-cremaster adenoviral dwelling time

For the incubation time course experiments, cremaster tumor bearing rats were administered 1 × 10^10 ^pfu's of Ad-CMV-Luc as previously described. The animals were then divided into three groups. In group I (n = 8), the muscle flaps were allowed to incubate for 5 minutes, in group II (n = 8) the muscle flaps were allowed to incubate for 30 minutes and in group III (n = 8) the muscle flaps were allowed to incubate for 1 hour. After the various times had elapsed, the vascular clamps were released, circulation was re-established, the muscle flaps were processed as previously described and the animals were placed in warm cages for observation. Seventy two hours following intra-arterial Ad-CMV-Luc delivery, animals were sacrificed with an intraperitoneal overdose of sodium pentobarbital. Tumors were collected in 1.5 mL polypropylene tubes and luciferase assay was performed as described below. For luciferase activity determination, tumors were collected in 1.5 mL polypropylene tubes and resuspended in 200 μl of luciferase lysis buffer (Promega Inc., Madison, WI). Tumors were then lysed using a manual tissue homogenizer, protein concentration was determined using the Bradford method and luciferase assay was done as indicated by the manufacturer using a Turner Designs TD-20/20 luminometer (Turner Designs, Sunnyvale, CA). Controls consisted of negative untreated tumors (n = 8) and mock (n = 8) groups. The negative control group consisted of untreated tumors that were collected at day 13 post cell implantation and assayed for luciferase activity. The mock control groups consisted of tumors that at day 10 after cell implantation were infused with viral preservation media, the cremasters were sutured as previously described and collected 72 hours post mock transfection for luciferase assay.

## Competing interests

The author(s) declare that they have no competing interests.

## Authors' contributions

GC: Conceptualized the model, carried out the adenoviral methodologies, performed the animal surgeries, processed the tissue, carried out the luciferase assays and wrote the manuscript. SLP: contributed with animal surgeries and aided in tissue processing, MS: participated with revising the manuscript, PD, participated with the experimental design and revising the manuscript, CG: participated with the experimental design and revising the manuscript,
